# Computational Modeling of *O*-Linked Glycan Biosynthesis in CHO Cells

**DOI:** 10.3390/molecules27061766

**Published:** 2022-03-08

**Authors:** Thukaa Kouka, Sachiko Akase, Isami Sogabe, Chunsheng Jin, Niclas G. Karlsson, Kiyoko F. Aoki-Kinoshita

**Affiliations:** 1 Department of Bioinformatics, Graduate School of Engineering, Soka University, Tokyo 192-8577, Japan; sachiko.akase@gmail.com (S.A.); e20m5908@soka-u.jp (I.S.); 2 Department of Cardiology, Keio University School of Medicine, Tokyo 160-8582, Japan; 3 Proteomics Core Facility at Sahlgrenska Academy, University of Gothenburg, 405 30 Gothenburg, Sweden; chunsheng.jin@medkem.gu.se; 4 Department of Life Sciences and Health, Faculty of Health Sciences, Oslo Metropolitan University, 0167 Oslo, Norway; niclaska@oslomet.no; 5 Glycan & Life Systems Integration Center (GaLSIC), Soka University, Tokyo 192-8577, Japan

**Keywords:** glycoinformatics, computational biology, *O*-linked glycans, glycan biosynthesis, systems biology

## Abstract

Glycan biosynthesis simulation research has progressed remarkably since 1997, when the first mathematical model for *N*-glycan biosynthesis was proposed. An *O*-glycan model has also been developed to predict *O*-glycan biosynthesis pathways in both forward and reverse directions. In this work, we started with a set of *O*-glycan profiles of CHO cells transiently transfected with various combinations of glycosyltransferases. The aim was to develop a model that encapsulated all the enzymes in the CHO transfected cell lines. Due to computational power restrictions, we were forced to focus on a smaller set of glycan profiles, where we were able to propose an optimized set of kinetics parameters for each enzyme in the model. Using this optimized model we showed that the abundance of more processed glycans could be simulated compared to observed abundance, while predicting the abundance of glycans earlier in the pathway was less accurate. The data generated show that for the accurate prediction of *O*-linked glycosylation, additional factors need to be incorporated into the model to better reflect the experimental conditions.

## 1. Introduction

The value of recombinant proteins as a main therapeutic approach, where human-like proteins are produced in host cells, has been increasing. The pharmacokinetics of these proteins in the human body vary according not only to the amino acid sequence, but also the glycans attached to the protein during the synthesis process. The enzyme-mediated process of adding glycans to a protein is a crucial post-translational modification (PTM) for its localization and trafficking, protein solubility, antigenicity, and cell–cell interaction [1,2,3,4]. For that reason, researchers have been studying the glycosylation process in order to understand and predict the produced glycan profiles of a protein in different biological systems.

One approach is computational modeling of the glycosylation process and studying the effect of changing variables on the final glycan profiles. The first mathematical model for *N*-glycan biosynthesis was developed in 1997 by Umana and Bailey (UB1997), simulating the initial stages of *N*-glycan biosynthesis from a single core structure [5]. Their model was built on the basic assumption that the four well-mixed compartments of the Golgi apparatus contain a set of eight glycosyltransferases. These enzymes catalyzed 33 reactions and generated 33 different oligosaccharide species of *N*-linked glycans attached to a specified protein. The model parameters were estimated from the literature describing Chinese Hamster Ovary (CHO) cells. The *N*-glycan profile predicted by the model was close to that of recombinant proteins produced by CHO cells. The UB1997 model laid the foundation for *N*-glycan modeling and was thereafter extended to many models.

In 2005, Krambeck and Betenbaugh extended UB1997 to include additional galactosylation, fucosylation, extension by adding *N*-acetyllactosamine repeats, and sialylation [6]. This KB2005 model generated 7565 oligosaccharide structures in a network of 22,871 reactions in terms of 11 enzymes and 20 reaction rules. The values of the model parameters were either derived from the UB1997 model or literature, or were adjusted in order to match the experimental *N*-glycan profiles in CHO cells. This model was further expanded and adjusted in 2009 by Krambeck et al. to estimate human *N*-glycans [7]. The KB2009 model included not only 19 enzymes with 26 reaction rules, but also a cutoff rule to stop the reactions after a specified molecular mass was reached. In 2017, another important model was developed by Krambeck and others, which successfully predicted the *N*-linked glycoform profiles of ten CHO cell lines, including one wild type and nine mutants [8].

By comparison, only a limited number of models were designed to simulate *O*-glycan synthesis. In 2008, Liu and co-workers modeled the first *O*-glycosylation reaction network through “object-oriented programming” [9]. The model started from core 2 structure Galβ1-3 (GlcNAcβ1-6) GalNAcol as the initial structure, while sialyl Lewis-X (sLex), a core 2 based *O*-linked glycan, was the only final product. In addition to core 2 and sLex, the reaction network contained another eighteen “intermediate” structures involved in 28 reactions. This model was fitted via sensitivity analysis to predict levels of sLex on (P-selectin glycoprotein ligand 1) PSGL-1 close to experimental data. On the other hand, McDonald et al. utilized a different methodology to develop their *O*-glycosylation network prediction software, called “*O*-Glycologue” [10]. This prediction tool included 25 enzymes and generated over 13 million possible unique structures in over 34 million reactions. *O*-Glycologue also has a feature to predict the synthesis pathway of any structure of the user’s choice. This feature traverses the reaction network in reverse starting from the end product, removing a monosaccharide with each reaction until it reaches the initial structure, a non-glycosylated Ser/Thr. This model did not include enzyme kinetics or mathematical modeling of the reactions. While the UB1997 model promoted *N*-glycan modeling and several models were built based on it, the lack of well-established studies modeling the kinetics of *O*-glycans’ synthesis slowed the advancement of *O*-glycan modeling.

In this study, we explore different methodologies for building a computational model of *O*-linked mucin-type glycan biosynthesis in CHO cells. We construct our models based on a set of experimental data in which the biological system of a cell line is modified by transiently transfecting the cells with different combinations of glycosyltransferases. Thereafter, the *O*-glycoform profile for each system is mapped and identified. We attempt to simulate these conditions and predict the subsequent glycan profile.

## 2. Results

### 2.1. Glycan Profiles Modeled in this Study

The experimental data utilized to build this model are based on our previous experiments on mapping the mucin-type *O*-glycan profile in CHO-K1 cells [11,12]. In total, 25 different combinations of glycosyltransferases were transiently expressed in CHO cells together with PSGL-1. Then, the mucin-type *O*-glycan profile was mapped for each cell line (Supplementary Materials). The recombinant protein PSGL-1 fused with the Fc portion of mouse immunoglobulin G2b (PSGL-1/mIgG2b) has more than 53 sites for mucin-type *O*-glycosylation [13]. The glycosyltransferases transiently expressed in CHO cells are shown in Table 1 [11,12].

Previous studies showed that the intensities of negatively charged glycan structures containing negatively charged residues, such as NeuAc, NeuGc, and/or sulfate, have a tendency to show higher signal in mass spectrometry studies [20]. Therefore, the intensities in our data were adjusted by a factor of 0.3 for singly charged glycans, and by a factor of 0.4 for doubly charged glycans. Consequently, the structures’ abundances across a glycan profile were recalculated.

### 2.2. Building the Models

**Figure 1:**
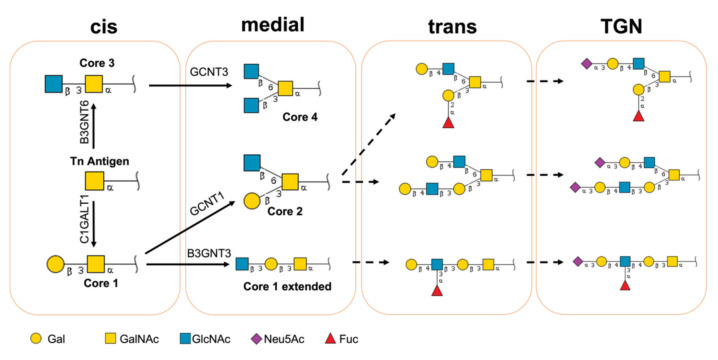
Mucin-type *O*-glycan biosynthesis pathways. Structures are depicted using the Symbol Nomenclature for Glycans (NSFG) symbol nomenclature (yellow square = GalNAc, yellow circle = Gal, blue square = GlcNAc, red triangle = Fuc, purple diamond = sialic acid).

The biosynthesis of GalNAc *O*-glycans is initiated in the Golgi apparatus, and carried out as the protein passes through the stacks of the Golgi apparatus and interacts with its content of glycosyltransferases (Figure 1) [21,22]. The Golgi apparatus stacks are functionally compartmentalized into the cis-Golgi network (CGN), cis-cisternae, medial-cisternae, trans-cisternae, and the trans-Golgi network (TGN) [23]. The glycosyltransferases’ distribution throughout the Golgi apparatus is not homogenous. Each compartment has a distinct composition of glycosyltransferases that reflects the sequence in which glycans are added to the proteins [24]. The cis and medial compartments contain core synthesizing enzymes, the medial and trans compartments mainly contain elongating glycosyltransferases, while capping and terminating enzymes are concentrated in trans and TGN compartments [25]. The biosynthesis of mucin-type glycans starts when an *N*-acetylgalactosamine (GalNAc) is attached to a polypeptide in the cis compartment [22,25]. A GalNAc linked to Ser/Thr is called a “Tn antigen”, and this reaction is catalyzed by a large family of GalNAc transferases [21]; however, it was not included in our model, since it necessitates considering the structure of the protein and the accessibility of the glycosylation sites. The Tn antigen is the initial structure in our model. This structure can be extended into one of the four cores (cores 1–4). The addition of a galactose (Gal) to the third carbon of GalNAc via a β-linkage forms “core 1 or T antigen”. This reaction is catalyzed by glycoprotein-*N*-acetylgalactosamine 3-beta-galactosyltransferase (C1GalT1) [26]. Competing with C1GalT1 for the same substrate (Tn Antigen) is beta-1,3-*N*-acetylglucosaminyltransferase (B3GNT6), which adds an *N*-acetylglucosamine (GlcNAc) at the same location to form “core 3” [27]. “Core 2” and “core 4” are formed by branching “core 1” and “core 3” via the attachment of a GlcNAc to the sixth carbon atom of the GalNAc, which is mediated by GCNT1 and/or GCNT3 (beta-1,6-*N*-acetylglucosaminyltransferase) [28,29]. All four cores are subsequently extended with LacNAc repeats, and are sulfated and/or sialylated before leaving the Golgi [21,22,25].

Considering that only transiently transfected glycosyltransferases were known, we computationally explored the reactions and glycosyltransferases required to produce the glycan structures found in each experiment. In total, 20 different enzymes were found to be active in producing all glycans, 12 of which were known transfected enzymes (Table 2). In case a family of enzymes catalyzed the same reaction, it was considered one enzyme for simplification reasons, as in B3GNT(s), B4GALT(s), ST3GAL(s), and ST6GALNAC(s). Four of the enzymes were *O*-glycan core synthesizing enzymes (C1GALT1, GCNT1/3, and B3GNT6). The list also included four fucosyltransferases (FUT2, 3, 4 and 7), three sialyltransferases (ST3GAL, ST6GAL1, and ST6GALNAC) and a sulfotransferase (CHST4). Six of the enzymes (B3GNT, B3GNT3, CHST4, GCNT1, ST3GAL, and ST6GALNAC) reacted with more than one substrate; therefore, multiple reaction rules were considered for these enzymes, and were differentiated by adding a small letter to the enzyme name. B3GNT_a and B3GNT_b are two reactions catalyzed by B3GNT(s). Our models included 30 reaction rules for 20 enzymes. The glycosyltransferases, their substrates, and the reactions they catalyze were derived from KEGG, and are listed in the Supplementary Materials [30].

**Figure 2:**
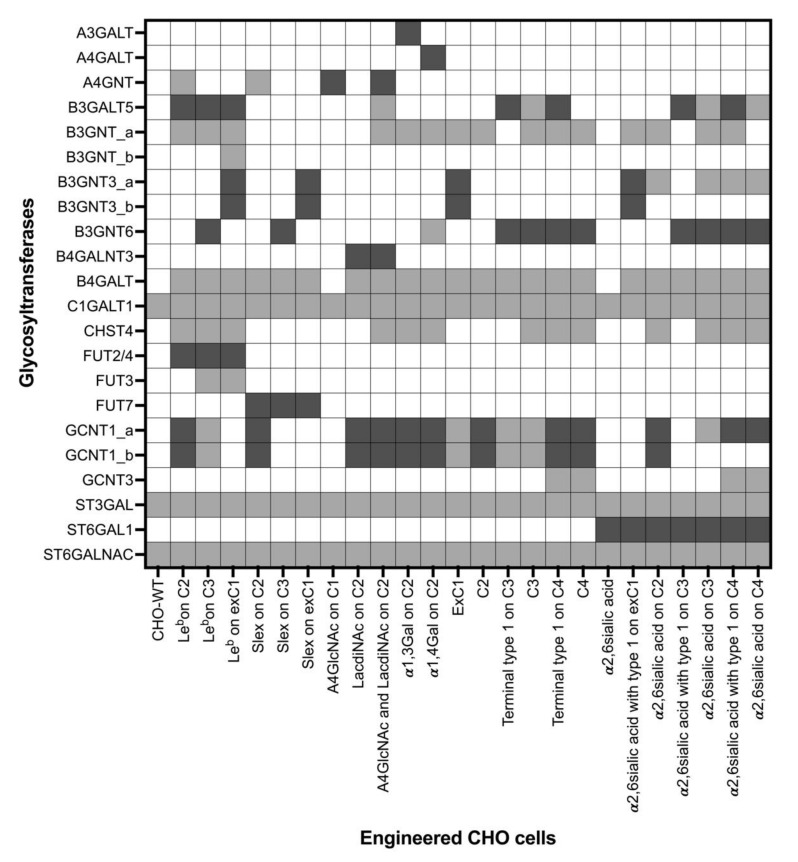
Glycosyltransferases included in modeling the *O*-glycan profiles of the 25 CHO cell lines. Dark grey represents the glycosyltransferases transiently expressed in the cells. Light grey represents the reactions of the glycosyltransferases required to produce a glycan profile. White cells represent enzymes that were neither transfected nor required for the glycan profile synthesis. C1: core 1; exC1: extended core 1; C2: core 2; C3: core 3; C4: core 4. “_a, _b” represents different reactions (reaction rules) catalyzed by a glycosyltransferase.

Glycosylation reaction networks were generated computationally per experiment and later incorporated into the mathematical model. The model of an engineered CHO cell line included the glycosyltransferases transiently expressed in the engineered cells and the glycosyltransferases required for the reaction network of that model to pro-duce the final glycan profile (Figure 2). In case an enzyme catalyzed two different re-actions, the model included the reaction rule included in the reaction network of that model.

To modulate the biosynthesis of mucin-type *O*-glycans, we designed a four-compartment model: “cis”, “medial”, “trans”, and “TGN”. The four compartments were set at a similar size and each compartment was assumed to be homogenous [5,6]. The glycosyltransferases were distributed across the four compartments depending on their role and order in the biosynthesis pathway. The protein residence time in a compartment was 5.56 min, during which the protein interacted with the glycosyltransfer-ases in that compartment following the modified Michaelis–Menten kinetics equation used in previous *N*-glycosylation models [6,7,8]. Then, it was transported to the next compartment following the Golgi mathematical model used in the Umana and Baily model (1997) [5]. After 22.24 min, the simulation ended, and the concentrations of structures accumulated in the TGN compartment were calculated. The sugar donor concentrations across the Golgi shown in Table 3 were based on previous works by Krambeck et al. [7,8]. The PAP_S initial concentration in Golgi was estimated based on Krambeck 2005 [6,31]. The CMP_NeuGc donor concentration was assumed to be simi-lar to CMP_NeuAc. The nucleotide-sugar donors in previous models were distributed equally across the compartments; however, in our model, this was limited to the com-partments where they donated the sugar, which was based on the enzyme distribution.

**Figure 3:**
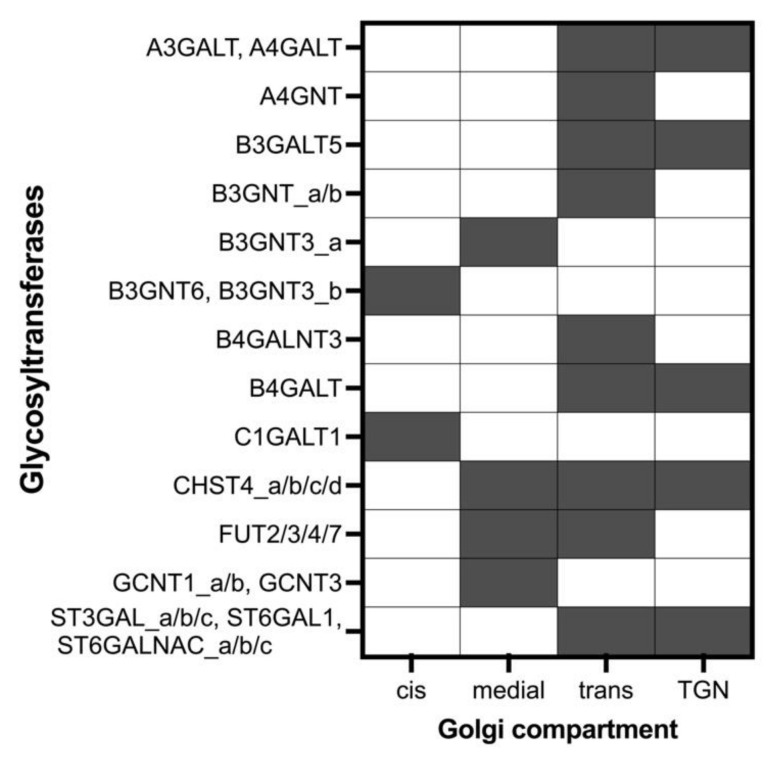
Glycosyltransferase localization across the four Golgi compartments. Filled cells represent the presence of the enzymes in a compartment, while white cells represent enzymes absence. “_a/b/c/d” represent different reactions (reaction rules) for the same enzyme.

The distribution of the enzymes across Golgi compartments shown in Figure 3 was set based on the reactions each enzyme catalyzes (core synthesis, elongation, capping etc.), and the localization of its substrates and the enzymes competing for that sub-strate. The core 1 and core 3 synthesizing enzymes compete for Tn Antigen and were localized in the “cis” compartment. Core 2 and core 4 synthesizing enzymes were as-sumed to peak in the second compartment. The elongating and capping glycosyltrans-ferases were localized in the last two compartments of trans and TGN. Fucosyltrans-ferases were distributed in the medial and trans compartments, while CHST4 was dis-tributed over all the compartments except cis.

### 2.3. Parameter Estimation and Simulations

**Figure 4:**
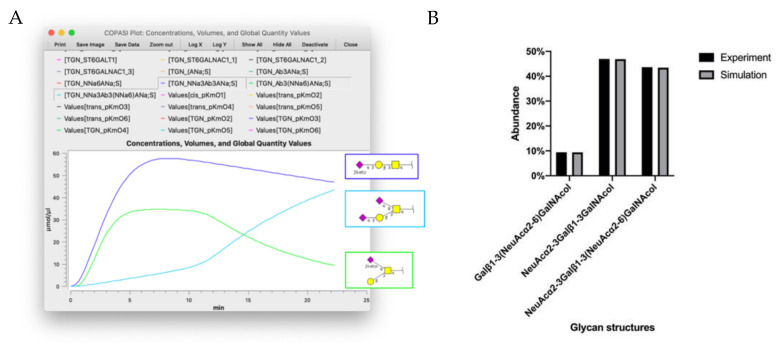
Simulation results of the CHO-WT cell line. (**A**) The simulation results in COPASI shows the glycan structures and their abundances in the last compartment “TGN”. (**B**) The abundance of each predicted structure at the end of the simulation compared to the concentrations measured experimentally. Structures are depicted using the Symbol Nomenclature for Glycans (NSFG) symbol nomenclature (yellow square = GalNAc, yellow circle = Gal, purple diamond = sialic acid).

The mathematical model based on a modified Michaelis–Menten kinetics equation requires enzyme concentrations, nucleotide-sugar donor concentrations and the kinetic parameters K*_m_*, K*_md_*, and K*_f_* for each enzyme. Nucleotide-sugar donor concentrations and enzyme localizations are illustrated in Figure 3 and Table 3. However, finding the rest of the parameters in the literature on CHO was challenging. Therefore, the tool Parameter Estimation in COPASI was utilized to find the initial values of the parameters to run time-course simulations and compare the resulting glycan profiles with the experimental glycan profiles. This process was computationally demanding; therefore, only experiments with a small glycan profile were modeled. The models “CHO” and “CHO-WT” model the mucin-type *O*-glycan profile in non-modified CHO cells. The glycan profile for these cells included three glycan structures, shown in Figure 4: “Galβ1-3(NeuAcα2-6)GalNAcol”, “NeuAcα2-3Galβ1-3GalNAcol”, and “NeuAcα2-3Galβ1-3(NeuAcα2-6)GalNAcol”. The simulation results in this model are shown in Figure 4. The line chart (Figure 4A) shows the change in concentration of the three main glycan structures in the TGN over the course of the simulation. Comparing the glycan profile predicted by the model and the experimental glycan profile in Figure 4B shows that the two profiles were almost identical. This indicates that using Parameter Estimation is a useful tool to generate possible values for our models’ parameters. However, running the same process for larger models proved to be challenging due to the currently available computing power.

**Figure 5:**
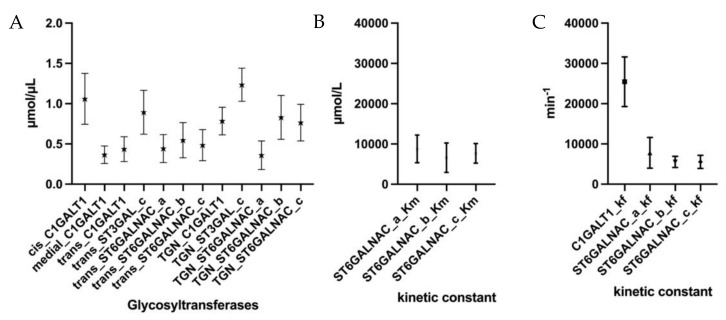
The 20 sets of parameters estimated for the CHO-WT model. (**A**) Estimated concentrations of the C1GALT1, ST3GAL, and ST6GALNAC in each compartment of Golgi. (**B**) Mean estimated K*m* values for ST6GALNAC. _a/b/c represents the three reactions catalyzed by the enzymes. (**C**) Mean estimated K*_f_* values for C1GALT1 and three reactions catalyzed by ST6GALNAC. Values are reported as mean with SEM.

To validate these results, the parameter estimation and time-course simulation were repeated 20 times. The simulation results are identical to the experimental data; however, the generated parameters were found to vary, as shown in Figure 5. While there is wide variance in most of the parameters, such as the K*_f_* value of C1GALT1 with a standard error of mean over 6000 min^−1^, other K*_f_* values were less deviating, with standard errors of mean of less than 1700 min^−1^ for ST6GALNAC_b and ST6GALNAC_c. However, further investigation is needed.

As discussed earlier, applying parameter estimation in one experiment gave satisfactory simulation results, but the parameter values changed during each iteration. This indicates that individual parameter values were not that crucial when a limited number of reactions were considered, as in the CHO-WT model. However, this created issues down the line when expanding this to larger experimental datasets, where we had 20 transferases generating a spectrum of parameters, generating a wider spread of the simulated abundance of individual structures. The ultimate goal would be to find a single solution (a set of values for the parameters) that is applicable to all the models, and that results in satisfactory glycan profile prediction for all the models. From there, we considered combining all the models into one large model and applying parameter estimation to this model. To achieve this, we would need increased computing power, since this was not achievable in the computers available in our institute.

**Figure 6:**
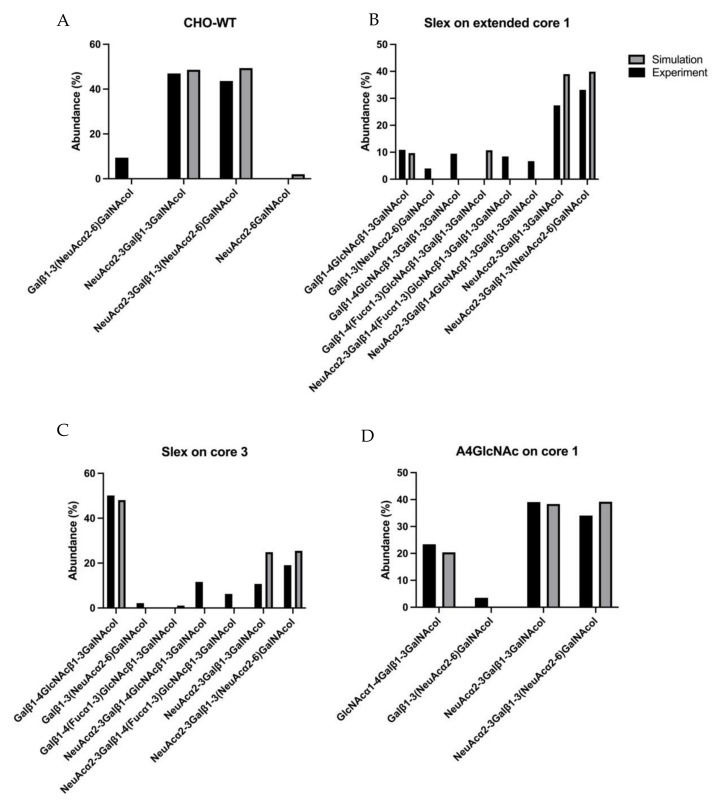
Predicted *O*-glycan profiles of four-experiment model. The abundance of predicted structures at the end of each model simulation is compared to the abundance measured experi-mentally. (**A**) CHO-WT model. (**B**) sLeX on extended Core 1 model. (**C**) sLeX on Core 3 model. (**D**) A4GlcNAc on Core1 model.

As an alternative approach to the computationally demanding large model, we designed a minimized model of a sample of relatively simple glycan profiles from four cell lines. Due to the limited number of glycans in their profiles, the following experiments were chosen: CHO-WT, Slex on Core 3, Slex on extended core 1, and A4GlcNAc on core 1. In this complex model, all the parameters in the four experiments were estimated simultaneously, then time-course simulations for each experiment were conducted separately. The calculation time for this model was about 4 h. A comparison of the simulation results with experimental results is illustrated in Figure 6, where the glycan profiles for each experiment are labeled with their experiment names. In CHO-WT and A4GLcNAc on core 1, the small number of glycans in these sets correlated well with the experimental data (Figure 6A,D). However, the simulation results for CHO-WT were less accurate when the parameters were estimated in the four-experiment model compared to the one-experiment model (Figure 4B and Figure 6A). On the other hand, there were larger disparities in the experiments generating Slex on core 3 and Slex on extended core 1. While Slex on core 1 overestimated the two most abundant glycans, it underestimated the abundances of NeuAcα2-3Galβ1-4(Fucα1-3)GlcNAcβ1-3Galβ1-3GalNAca1- and Galβ1-4GlcNAcβ1-3GalNAca1-. The disparities were even greater in Slex on core 3, where the levels of three structures were underestimated, including the final product NeuAcα2-3Galβ1-4(Fucα1-3)GlcNAcβ1-3GalNAcol. However, it predicted correctly that the galactosylated core 3 would be the dominating structure produced after transfection.

## 3. Discussion

Even though there were many technical challenges involved in performing large model simulations in this work, we were able to gain new insight into various aspects of *O*-glycan biosynthesis enzymes. We obtained estimations of the kinetic constants and concentrations for each reaction in a single experiment that gave simulation results close to the experimental data (Figure 4 and Figure 5).

On the other hand, Figure 6 illustrates that there were discrepancies between different experimental conditions when different enzymes were transiently expressed in separate experiments but on the same cell line. While our simulations were able to predict experimental glycome profiles per experiment, a combined model could not accurately predict all glycome profile across all experiments accurately. This might be due to the experimental factors involved that still need to be considered. Another possible factor that was not included in our model is simulating the initial step of the mucin-type *O*-linked biosynthesis, performed by the GalNAc-polypeptide transferases. In general, our simulation results could predict the more highly processed glycans more accurately than the structures that are synthesized earlier in the pathway. Indeed, it has been reported that the neutralization of pH in the Golgi apparatus causes glycosyltransferases to redistribute, causing the premature termination/elongation of *O*-linked glycans [32]. This suggests that the Golgi distributions of glycosyltransferases are not static. During cell culturing, increased confluence would cause some cells to experience hypoxia, affecting the cellular level of sugar nucleotides and the glycosylation machinery [33]. Therefore, it could very well be the case that such factors as the altered pH of the Golgi and hypoxia could cause the redistribution of glycosyltransferases during extended cell culturing. Altered sugar nucleotide levels would also need to be incorporated into the simulation of cell culture models to be able to make more accurate predictions about glycan biosynthesis. In addition, there is the possibility that transiently transfecting additional glycosyltransferases in CHO cells subsequently redistribute glycosyltransferases and nucleotide transporters in the Golgi compartments. The multi-assembly of glycosyltransferases and nucleotide transporters in Golgi has been shown for *N*-linked biosynthesis [34]. Whether *O*-linked glycoenzymes in the Golgi are also assembled in a similar manner, and how these assemblies are restructured as additional transferases are introduced, are unknown. Hence, for the accurate simulation of glycosylation, our initial approach of identifying a single set of parameters that could fit into one model for all 20 enzymes will have its limitations.

## 4. Materials and Methods

Engineering the CHO cell lines included the transient transfection of cells with different combinations of glycosyltransferases for each cell line, as described in our previous work [10,11].

The computer models were generated in extendable markup language “XML”, using our in-house developed tool “GlycoSim” [35,36]. Linear code was used to describe the glycan structures and the reaction rules. The localization of an enzyme in the four compartments was described as 0 and 1. Models were then imported into the complex pathway simulator “COPASI”, where the parameter estimations and time simulations were processed [37]. In COPASI, the volume of each compartment was changed to 2.5 µL and the initial glycan concentration was changed to 100 µmol/µL.

For the parameter estimation, we used the Evolution Strategy (SRES) method. Experimental data were matched to the concentration of the glycan structure at the TGN compartment. The weight method was set to standard deviation. Parameters to be estimated were added and cross-referenced to their relevant experiment. The lower and upper bounds were set to 10^−6^ and 10^5^, respectively. In the case of the four-experiment model, we updated the model with the estimated parameters then saved it in four separate files relevant to the experiments. In each model, we set the concentrations of enzymes that were not relevant to the experiments to 0 µmol/µL. The time-course simulations deterministic (LSODA) method was used. The results were analyzed using GraphPad Prism 9 for macOS, GraphPad Software, San Diego, California USA, www.graphpad.com (accessed on 24 February 2022).

## Figures and Tables

**Table 1:** 25 CHO cells transfected with combinations of glycosyltransferases. CHO/CHO-WT: CHO-K1; C1: core 1; exC1: extended core 1; C2: core 2; C3: core 3; C4: core4; type 1: terminal type 1.

Engineered CHO Cells’ Names	Transfected Glycosyltransferases	Reference
CHO/CHO-WT	-	[11,14]
Le^b^ on C2	GCNT1, B3GalT5, FUT2, FUT4	[15,16], Unpublished data
Le^b^ on C3	B3GNT6, B3GalT5, FUT2, FUT4	[15,16], Unpublished data
Le^b^ on exC1	B3GNT3, B3GalT5, FUT2, FUT4	[15,16], Unpublished data
Slex on C2	GCNT1, FUT7	[15], Unpublished data
Slex on C3	B3GNT6, FUT7	[15], Unpublished data
Slex on exC1	B3GNT3, FUT7	[15], Unpublished data
A4GlcNAc on C1	A4GNT	Unpublished data
LacdiNAc on C2	GCNT1, B4GALNT	[17]
A4GlcNAc and LacdiNAc on C2	GCNT1, A4GNT, B4GALNT	Unpublished data
α1,3Gal on C2	GCNT1, A3GALT	[18]
α1,4Gal on C2	GCNT1, A4GALT	[18]
ExC1	B3GNT3	[11]
C2	GCNT1	[11,19]
Terminal type 1 on C3	B3GNT6, B3GalT5	[12,16]
Terminal type 2 on C3	B3GNT6	[11,16]
Terminal type 1 on C4	GCNT1, B3GNT6, B3GalT5	[12,16]
C4	GCNT1, B3GNT6	[12]
α2,6sialic acid	ST6GAL1	[11,12]
α2,6sialic acid with type 1 on exC1	ST6GAL1, B3GNT3	[11,12]
α2,6sialic acid on C2	ST6GAL1, GCNT1	[11,12]
α2,6sialic acid with type 1 on C3	ST6GAL1, B3GNT6, B3GALT5	[12,16]
α2,6sialic acid on C3	ST6GAL1, B3GNT6	[11,12]
α2,6sialic acid with type 1 on C4	ST6GAL1, B3GNT3, GCNT1, B3GALT5	[12,16]
α2,6sialic acid on C4	ST6GAL1, B3GNT3, GCNT1	[12]

**Table 2:** Glycosyltransferases used in CHO *O*-glycan profile model.

Abbreviation	Glycosyltransferase	EC Number	Cores or Terminal Epitopes
A3GALT *	Alpha-1,3-galactosyltransferase	2.4.1.-	αGal
A4GALT *	Alpha-1,4-galactosyltransferase	2.4.1.228	P1 antigen
A4GNT *	Alpha-1,4-*N*-Acetylglucosaminyltransferase	2.4.1.-	α1,4GlcNAc
B3GALT5 *	Beta-1,3-galactosyltransferase 5	2.4.1.-	Type 1 chain
B3GNT	Beta-1,3-*N*-Acetylglucosaminyltransferase	2.4.1.149	
B3GNT3 *	Beta-1,3-*N*-Acetylglucosaminyltransferase 3	2.4.1.146	Extended core 1
B3GNT6 *	Beta-1,3-*N*-Acetylglucosaminyltransferase 6	2.4.1.147	Core 3
B4GALNT3 *	Beta-1,4-*N*-acetylgalactosaminyltransferase 3	2.4.1.244	LacdiNAc
B4GALT	BetBeta-1,4-galactosyltransferase	2.4.1.38	Type 2 chain
C1GALT1	Glycoprotein-*N*-acetylgalactosamine beta-1,3-galactosyltransferase	2.4.1.122	Core 1
CHST4	Carbohydrate sulfotransferase 4	2.8.2.-	6SGlcNAc
FUT2 *	Fucosyltransferase 2	2.4.1.-	α1,2Fuc
FUT3	Fucosyltransferase 3	2.4.1.65	α1,3/4Fuc
FUT4 *	Fucosyltransferase 4	2.4.1.	α1,3Fuc
FUT7 *	Fucosyltransferase 7	2.4.1.	α1,3Fuc
GCNT1 *	Beta-1,6-*N*-acetylglucosaminyltransferase 1	2.4.1.102	Core 2/4
GCNT3	Beta-1,6-*N*-acetylglucosaminyltransferase 3	2.4.1.148	Core 2/4
ST3GAL	Beta-galactoside alpha-2,3-sialyltransferase	2.4.99.4, 2.4.99.6	α2,3Sia
ST6GAL1 *	Beta-galactoside alpha-2,6-sialyltransferase 1	2.4.99.1	α2,6Sia
ST6GALNAC	ST6 *N*-acetylgalactosaminide alpha-2,6-sialyltransferase	2.4.99.3	α2,6Sia

* Glycosyltransferases transiently expressed in one or more experiments. EC number: Enzyme Commission number.

**Table 3:** Donor concentrations in µM and distribution across the Golgi compartments.

Donor	cis	Medial	Trans	TGN
CMP_NeuAc	0	0	3000	3000
CMP_NeuGc	0	0	3000	3000
GDP_Fuc	0	5000	5000	0
UDP_GlcNAc	9143	9143	9143	0
UDP_Gal	3810	0	3810	3810
UDP_GalNAc	0	0	3000	0
PAP_S	0	920	920	920

## Data Availability

The data presented in this study are available from [computational modeling of *O*-linked glycans biosynthesis in CHO cells].

## Supplementary Materials

The following supporting information can be downloaded at: https://www.mdpi.com/article/10.3390/molecules27061766/s1.

## Sample Availability

Samples of the compounds are available from the authors.
