# Supplementary material for: Computational Modeling of O-Linked Glycan Biosynthesis in CHO Cells
Source: Molecules. 2022 Mar 8;27(6):1766. doi: 10.3390/molecules27061766 (PMC8950484; doi:10.3390/molecules27061766)
Supplement: Supplementary file 1 [file molecules-27-01766-s001.zip › supp figs.pdf]

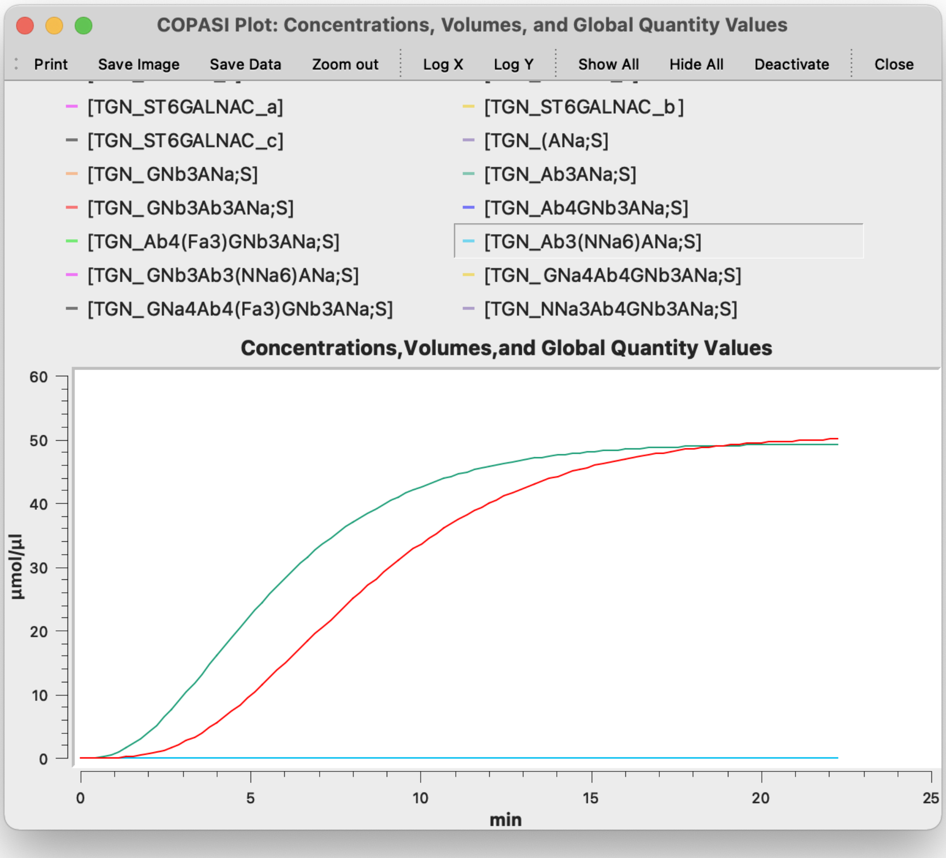

Figure S1. Simulation results of the CHO-WT in the four-experiment model**.** The simulation results in COPASI shows the glycan structures and their abundances in the last compartment "TGN".

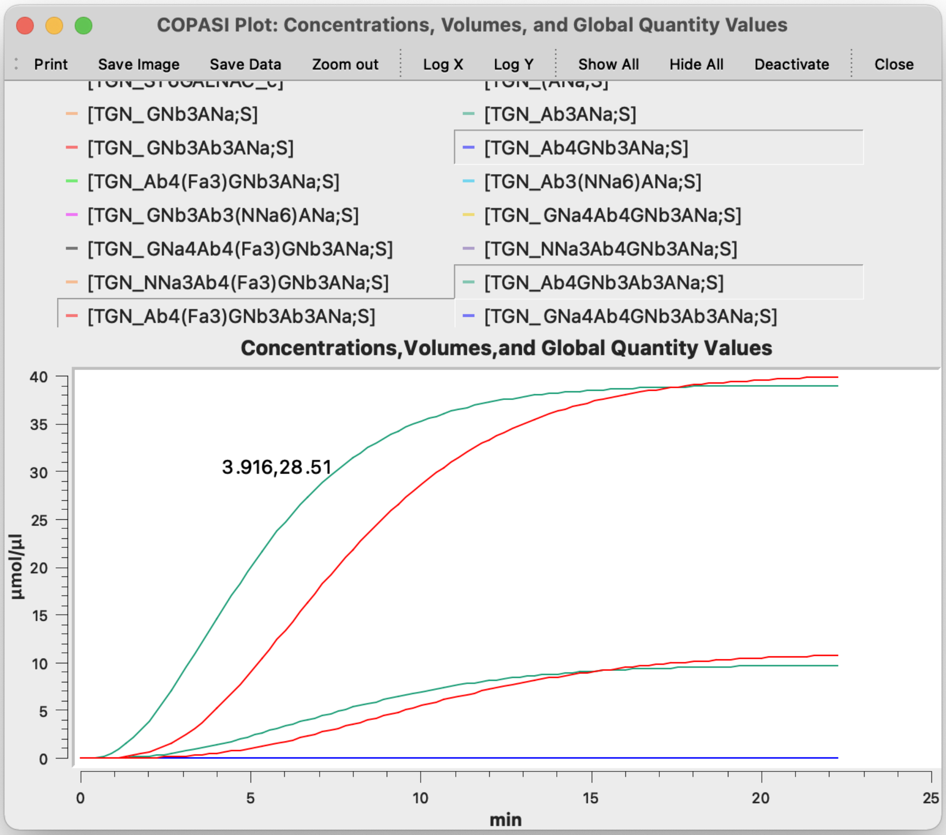

Figure S2. Simulation results of the sLeX on extended Core 1 in the four-experiment model**.** The simulation results in COPASI shows the glycan structures and their abundances in the last compartment "TGN".

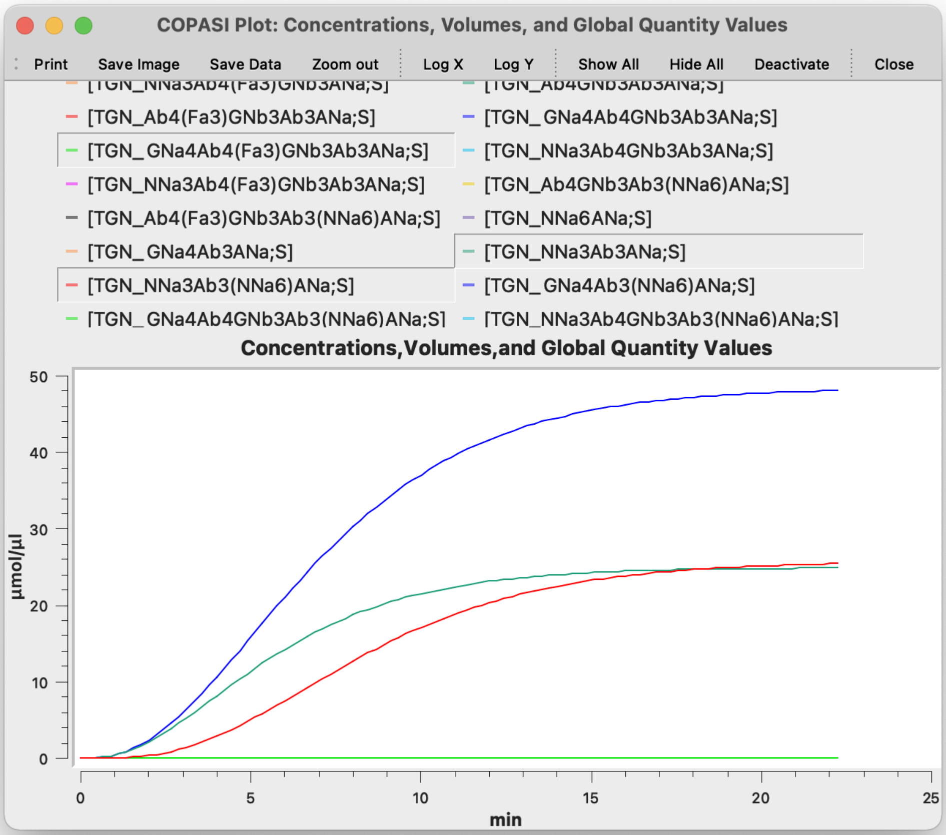

Figure S3. Simulation results of the sLeX on Core 3 in the four-experiment model. The simulation results in COPASI shows the glycan structures and their abundances in the last compartment "TGN".

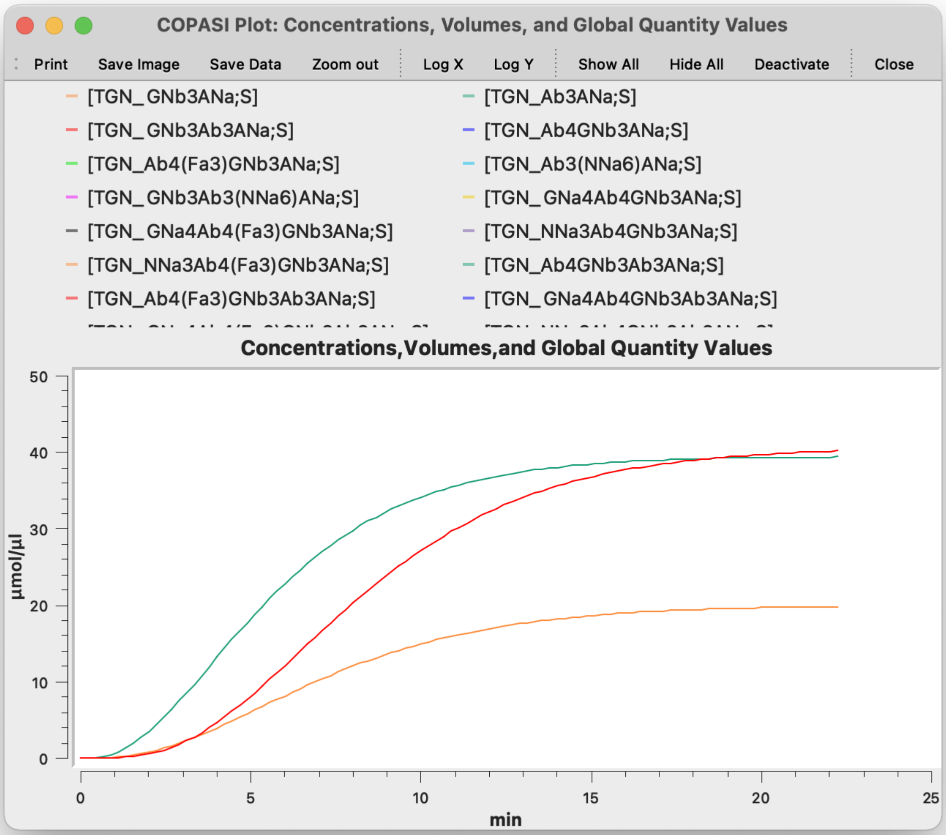

Figure S4. Simulation results of the A4GlcNAc on Core 1in the four-experiment model. The simulation results in COPASI shows the glycan structures and their abundances in the last compartment "TGN".
